# μ-Opioid receptors negatively regulate striatal dopamine D_1_ receptor signaling through heteromerization

**DOI:** 10.21203/rs.3.rs-10628538/v1

**Published:** 2026-09-30

**Authors:** Arantzazu Alonso-Carrasco, William N. Sánchez, Natàlia Llopart, Marta Valle-León, Marc Ciruela-Jardí, César Quiroz, Ning-Sheng Cai, Cèlia Rodríguez-Casas, Christopher J. Earley, Estefanía Moreno, Verònica Casadó-Anguera, Leonardo Pardo, Vicent Casadó, Sergi Ferré

**Affiliations:** 1 Department of Biochemistry and Molecular Biomedicine, Faculty of Biology and Institute of Biomedicine, University of Barcelona, Barcelona, Spain,; 2 Integrative Neurobiology Section, National Institute on Drug Abuse, Intramural Research Program, National Institutes of Health, Baltimore, MD,; 3 Laboratory of Computational Medicine, Biostatistics Unit, Faculty of Medicine, Autonomous University of Barcelona, Barcelona, Spain,; 4 Center for Restless Legs Study, Department of Neurology, Johns Hopkins University, Baltimore, MD.

## Abstract

Integrating biophysical, computational, *in situ*, and *in vivo* approaches with selective heteromer-disrupting transmembrane peptides, this study demonstrates that μ-opioid receptors (MORs) and dopamine D_1_ receptors (D_1_Rs) assemble into functional heteromers. Furthermore, real-time *in vivo* fiber photometry using a genetically encoded cAMP biosensor directly demonstrates that MORs regulate striatal D_1_Rs via heteromerization in the intact mouse brain. Our findings support a tetrameric structure composed of Gi-coupled MOR and Gs-coupled D_1_R homodimers, which enables both canonical Gi-Gs antagonistic interaction at the level of adenylyl cyclase and receptor-level cross-antagonism, by which MOR agonists and antagonists counteract D_1_R agonist-induced signaling. Located predominantly within striosomal GABAergic projection neurons, MOR–D_1_R heteromers constitute mechanistically defined signaling units that integrate dopaminergic and opioid neurotransmission governing motor control and reward processing. Collectively, these findings establish MOR–D_1_R heteromers as a previously unrecognized therapeutic target for the treatment of opioid withdrawal-associated restlessness and restless legs syndrome.

## Introduction

Both the therapeutic effects and the undesired side effects of opioids are largely mediated by μ-opioid receptors (MORs). One of the highest densities of MORs in the brain is found in the striatum, specifically within the compartment known as striosomes (or patches), as opposed to the surrounding matrix compartment. Striosomes were originally described as “striatal islands of closely packed opioid receptors”^1^. In this compartment, MORs are predominantly expressed by striatal GABAergic projection neurons within dendritic domains closely apposed to dopaminergic terminals^2^. These neurons primarily express dopamine receptors of the D_1_ subtype (D_1_Rs)^3,4^.

Both the striosomal and matrix compartments contain GABAergic projection neurons that express either D_1_ or D_2_ dopamine receptors (D_1_Rs or D_2_Rs)^5^. These neuronal populations have classically been associated with the direct and indirect striatal efferent pathways of the basal ganglia, respectively, which connect the striatum to the main output nuclei of the basal ganglia: the substantia nigra pars reticulata (SNpr) and the internal segment of the globus pallidus (GPi; the entopeduncular nucleus, EPN, in rodents)^5^. The striatum serves as the principal input structure of the basal ganglia, where glutamatergic cortical and thalamic afferents, together with mesencephalic dopaminergic inputs, converge onto GABAergic projection neurons. These projection pathways ultimately influence thalamic glutamatergic neurons that project back to the cortex, thereby completing the cortico–striato–thalamo–cortical circuit^5^. However, this classical scheme primarily reflects the outputs of the matrix compartment. In contrast, striosomal MOR–D_1_R neurons project to dopaminergic neurons of the substantia nigra pars compacta (SNpc) and ventral tegmental area (VTA) and to glutamatergic GPi/EPN neurons that innervate the lateral habenula (LHb)^2,5–7^.

The striosomal MOR–D_1_R neurons have been proposed to play a central role in the behavioral effects of opioids, including their rewarding properties and, more recently, the restlessness associated with opioid withdrawal and restless legs syndrome (RLS)^8^. Clinically, evidence supporting the efficacy of opioids in RLS dates back to the 17th century^9^, and the restlessness observed during opioid withdrawal frequently meets the diagnostic criteria for RLS^10,11^. We therefore recently hypothesized that the restlessness of RLS and opioid withdrawal share the same neural mechanisms^8^. Experimentally, increased sensitivity of striosomal MOR–D_1_R neurons in promoting GABA release in the EPN was first demonstrated in mice following withdrawal from sustained morphine treatment^4^. More recently, we observed the same phenomenon in a brain iron–deficient mouse model of RLS. Specifically, we found that a low dose of a D_1_R agonist—ineffective in control animals—is sufficient to induce GABA release in the EPN, and that this effect is antagonized by a MOR agonist^12^. In the same study, we found that brain iron deficiency was associated with a significant decrease in striatal MOR mRNA expression, without significant changes in D_1_R expression^12^—a phenomenon that has also been observed experimentally following chronic opioid exposure^13,14^. Because D_1_Rs and MORs are coupled to stimulatory Gs and inhibitory Gi proteins, respectively, we hypothesized that the restlessness observed in RLS and opioid withdrawal results from the disinhibition of striatal D_1_R signaling due to MOR downregulation^12^. Demonstrating functional MOR–D_1_R antagonistic interactions in the striatum could provide key insights into the pathogenesis of these disorders and guide novel therapeutic strategies.

Accumulating evidence indicates that GPCR heteromerization can generate receptor complexes with distinct biochemical and pharmacological properties^15,16^. In previous studies, MORs and D_1_Rs were proposed to form functional heteromers both in mammalian transfected cells and in the striatum^17,18^. However, those studies focused on whether MOR–D_1_R heteromers mediate the ability of D_1_R ligands to modulate the binding and signaling properties of MOR ligands and only provided indirect evidence for their existence in striatal tissue^17,18^. Whether MOR–D_1_R heteromers exist as structurally defined complexes in the striatum and directly mediate the antagonistic control of D_1_R signaling by opioids remains to be demonstrated.

In this study, we aimed to provide unequivocal evidence for striatal MOR–D_1_R heteromers, determine the structural organization of the receptor complex, and establish whether these heteromers mediate the antagonistic effects of opioids on striatal D_1_R signaling. Using complementary biochemical, biophysical, computational, *in situ* and *in vivo* approaches, we demonstrate that MOR and D_1_R form functional heteromeric complexes in the striatum. Furthermore, we show that the structural integrity of these MOR–D_1_R heteromers is required for the antagonistic modulation of D_1_R signaling by MOR ligands.

## Results

### Quaternary structure of the Gi-MOR–Gs-D_1_R heteromer

Previous studies proposed that GPCR heteromers commonly form a tetrameric quaternary structure composed of two homodimers, each coupled to its preferred G protein^15^. These heterotetramers frequently appear to be constituted by an inhibitory Gi-coupled receptor and a stimulatory Gs-coupled receptor. The first aim of this study was to find evidence for a similar quaternary structure of the MOR–D_1_R heteromer. Bimolecular Luciferase Complementation (BiLC) experiments were performed with MOR and D_1_R separately fused to complementary halves of the luminescent protein *Renilla* Luciferase (MOR-nRLuc and D_1_R-cRLuc) in the presence and absence of MOR and D_1_R TM-peptides. These peptides are fused to the cell-penetrating HIV trans-activator of transcription (TAT) peptide (YGRKKRRQRRR) to allow their right orientation when inserted in the plasma membrane^19–21^. Of the eleven TAT-TM peptides tested, the bioluminescent signal was significantly reduced only with the TM4 and TM5 peptides from both the MOR and the D_1_R. (Fig. 1a), supporting the existence of a symmetrical TM4-TM5/TM5-TM4 MOR–D_1_R heteromeric interface (TM4/5 interface).

Previous studies have shown that MOR prefers a homodimeric structure at high physiological densities, featuring a symmetrical TM5-TM6/TM6-TM5 interface (TM5/6 interface) resolved by X-ray crystallography^22,23^. Computational analysis further indicates that this TM5/6 interface is structurally compatible with a simultaneous TM4/5 heteromeric interface (Fig. 1d). More recently, an investigation into the dynamic nature of GPCR oligomerization revealed that the intracellular C-terminal domain also plays a significant role in MOR dimerization^24^. We next evaluated the putative D_1_R homomeric interface using BiLC assays, both in the presence and absence of D_1_R TM-peptides. We could first show that bioluminescence of D_1_R-nRLuc/D_1_R-cRLuc was only reduced in the presence of TM4 and TM5 peptides, also indicating a TM4/TM5 homomeric interface (Fig. 1b). This shared interface with MOR would initially seem to preclude D_1_R from concurrently forming homomers and heteromers with MOR. However, when D_1_R-nRLuc and D_1_R-cRLuc were co-transfected with non-fused MOR, bioluminescence was only significantly decreased by TM4, TM5 and TM6 peptides of D_1_R (Fig. 1c). This suggests that the presence of MOR induces a structural shift in the D_1_R homomeric interface from a TM4/5 to a TM5/6 interface. Given the dynamic nature of GPCRs, different homo- and heterodimerization interfaces can potentially interconvert^25^ and can be influenced by ligand binding^26^, as well as by interactions with other GPCRs^22^. It is noteworthy that the TM4 peptide of D_1_R continues to influence D_1_R homodimerization in the presence of MOR (Fig. 1c), even though TM4 is not part of the TM5/6 homodimerization interface. This observation suggests the coexistence of a population of D_1_R homomers that do not associate with MOR and therefore retain their preferred TM4-TM5 dimerization interface (Fig. 1b). In conclusion, the experimental results indicate the MOR–D_1_R heteromer displays a quaternary structure in which homodimerization occurs via the TM5/6 interface and heteromerization occurs via TM4/5 MOR–D_1_R interface (Fig. 1d).

Comparable quaternary rearrangements have been previously documented for other GPCR heterotetramers^19,22,27–29^. In these cases, it was proposed that the outer protomer of one homodimer couples to a Gs protein, whereas the outer protomer of the partner homodimer couples to a Gi protein (Fig. 1e). However, in the proposed MOR–D1R heteromer, the TM4/5 heteromerization interface adopts a sufficiently distant conformation to accommodate the simultaneous coupling of one G protein to the inner protomer and a second G protein to the outer protomer of the partner homodimer (Fig. 1f). A clash between both G proteins prevents them from binding simultaneously to the inner protomers (not shown). Nevertheless, recent cryo-EM studies of the κ-opioid receptor homodimer, assembled through the TM1–TM7/TM7–TM1 interface, have shown that the greater separation between the two protomers permits the simultaneous binding of two G proteins to a single homodimer^30^. Molecular dynamics (MD) simulations reveal subtle rearrangements in the shape of the MOR–D_1_R heterotetramer that depend on the sites of G protein coupling **(Fig. S1)**.

### Canonical Gi-Gs antagonistic interaction at adenyl cyclase

These arrangements of G proteins in the MOR–D_1_R heterotetramer may facilitate the canonical Gi-Gs antagonistic interaction at adenylyl cyclase (AC), whereby activation of a Gi-coupled receptor counteracts the cyclic AMP (cAMP) formation triggered by a Gs-coupled receptor^15,31^. We previously demonstrated that this type of functional interaction between GPCRs depends on their heteromerization and is driven by intermolecular interactions between TM domains of AC and the heteromer^19^. By using synthetic TM-peptides corresponding to the TM sequences of the Gi-coupled D_2_R, the Gs-coupled adenosine A_2A_R receptor and AC, we confirmed that a D_2_R agonist’s ability to counteract A_2A_R-mediated cAMP formation relies entirely on the structural integrity of the A_2A_R–D_2_R heteromer and its direct interactions with AC^19^. Numerous other heteromers mediate this canonical Gi-Gs antagonistic interaction at AC, as shown for A_1_R–D_1_R, A_2A_R–cannabinoid CB_1_ receptor (CB_1_R), D_1_R–D_3_R, MOR–galanin Gal_1_ receptor (Gal_1_R) and MOR–corticotropin releasing factor CRF_1_ receptor (CRF_1_R) heteromers^32–34,22,27^. We termed these functional complexes G protein-coupled–effector macromolecular membrane assemblies (GEMMAs), and the canonical Gi-Gs antagonistic interaction at AC was labelled as type III allosterism in a GPCR heteromer^35^.

Accordingly, we next evaluated whether the MOR–D_1_R heterotetramer exhibits the canonical antagonistic interaction between Gi and Gs proteins at AC. Experiments on cAMP formation were performed in HEK-293T cells stably transfected with MORs and D_1_Rs. The D_1_R agonist SKF38393 (1 μM) promoted cAMP formation, while the endogenous MOR agonist endomorphin-1 (1 nM) counteracted cAMP formation induced by both forskolin (0.5 μM) and SKF38393 (Fig. 2a). The fact that the MOR agonist inhibited the cAMP-promoting effect of the D_1_R agonist, revealed a canonical Gi-Gs antagonist interaction. TM peptides were then used to determine whether the integrity of the MOR–D_1_R heteromer is required for the canonical Gi-Gs antagonistic interaction at AC. Simultaneous application of TM4 and TM5 peptides was necessary to significantly counteract the ability of endomorphin-1 to inhibit the effect of SKF38393 (Fig. 2b).

### Cross-antagonism of D_1_R signaling in the MOR–D_1_R heteromer

Cross-antagonism was also observed when evaluating the effect of antagonists in cAMP experiments **(Fig. a)**. Cross-antagonism is a hallmark of GPCR heteromers and is classified as Type I allosterism, in which a ligand binding to one protomer modulates the affinity or efficacy of a ligand at the neighboring protomer^35^. As expected, the MOR antagonist naloxone (1 μM) counteracted the inhibitory effect of the MOR agonist endomorphin-1 on forskolin-induced cAMP accumulation (antagonism). Remarkably, naloxone also significantly reduced the cAMP response elicited by the D_1_R agonist SKF38393 (cross-antagonism). Conversely, the D_1_R antagonist SCH23390 (1 μM) not only blocked SKF38393-induced cAMP accumulation (antagonism), but also the inhibitory effect of endomorphin-1 on forskolin-induced cAMP (cross-antagonism). TM peptides were subsequently used to assess whether the formation of the MOR–D_1_R heteromer is required for cross-antagonism, as previously demonstrated for the canonical Gi-Gs antagonistic interaction at AC. Application of only TM4 or TM5, but not TM6, peptides of the D_1_R or the MOR counteracted the ability of naloxone to inhibit D_1_R agonist-induced cAMP accumulation (Fig. 2c). Since the simultaneous application of both TM4 and TM5 peptides was required to significantly disrupt the canonical antagonistic interaction between Gi and Gs proteins at AC, it could be concluded that cross-antagonism is more sensitive to partial disruption of heteromers, whereas the canonical Gi-Gs antagonism requires more extensive destabilization before an effect becomes detectable.

To further evaluate this Type I allosterism, we used NanoLuc Binary Technology (NanoBiT) and complemented donor-acceptor resonance energy transfer (CODA-RET) assays. In a NanoBiT assay performed in HEK-293T cells, the SmBiT and LgBiT complementary fragments of NanoLuc luciferase are fused to the D_1_R and the α subunit of the Gs protein, respectively. The cells were also co-transfected with non-fused MOR. Addition of the D_1_R agonist SKF81297, by promoting receptor coupling to Gs, triggered the concentration-dependent reconstitution of SmBiT and LgBiT nanoluciferase bioluminescence (Fig. 3a). Addition of naloxone (100 nM) significantly decreased the efficacy (E_max_) of SKF81297 but not its potency (EC_50_), while the addition of the MOR agonist methadone (100 nM) did not modify the concentration-response curve of SKF38393 (Fig. 3a). CODA-RET assay, a variant of the BRET technique allows a more direct analysis of G protein coupling to a GPCR heteromer^22,27,29,36,37^. In this assay, two complementary halves of RLuc (nRLuc and cRLuc) are separately fused to two different GPCR protomers putatively able to dimerize, and YFP is fused to the α subunit of a G protein. Ligand-induced changes in CODA-RET measurements imply a successful complementation of nRLuc and cRLuc and, therefore, dimerization of the corresponding protomers. Moreover, it illustrates G protein activation driven by a GPCR heteromer. MOR fused to cRLuc (MOR-cRLuc) and D_1_R fused to nRLuc (D_1_R-nRLuc) were co-transfected with Gs fused to YFP (mVenus variant) and untagged Gβ1 and Gγ2 subunits in HEK-293T cells. Dopamine produced a clear concentration-response curve, indicative of an increased Gs coupling to the MOR–D_1_R heteromer (Fig. 3b). In agreement with the results from the Nano-BiT assay, naloxone, but not methadone or endomorphin-1 (1 μM), significantly decreased the efficacy (E_max_), but not the potency (EC_50_) of dopamine (Fig. 3b).

To determine whether similar interactions occur independently of G-protein signaling, we used a BRET assay in HEK-293T cells to analyze the effects of naloxone, methadone, and endomorphin-1 (10 μM) on dopamine-induced β-arrestin2 recruitment. Cells were co-transfected with D_1_R-YFP (Venus variant), β-arrestin2-RLuc, and non-fused MOR. Dopamine promoted a concentration-dependent β-arrestin2 recruitment which potency (EC_50_), but not efficacy (E_max_) was significantly decreased by naloxone, but not methadone or endomorphin-1 (Fig. 3c). Because MOR antagonists promote cross-antagonism of D_1_R agonist-induced G-protein coupling and β-arrestin recruitment, this mechanism likely plays a significant role in both G protein-dependent and -independent signaling.

Since antagonists do not initiate signaling on their own, cross-antagonism can only occur through direct receptor–receptor interactions. Because naloxone binding to the MOR counteracts SKF38393-induced D_1_R signaling, we propose that this effect is mediated when naloxone binds the inward-facing, inactive, Gi-unbound protomer of the MOR homodimer, and SKF38393 binds the inward-facing, active, Gs-bound protomer of the D_1_R homodimer (Fig. 4a). These two inward-facing protomers are proposed to interact directly through the TM4/5 MOR–D_1_R heteromeric interface. We suggest that the allosteric mechanism is mediated by the predicted TM contacts between receptor protomers^38^, which are restricted to the extracellular regions of TM4 and TM5 and to ICL2. Comparison of the naloxone- and morphine-bound MOR structures^39,40^ reveals a subtle conformational difference at the top of TM5 (Fig. 4b) and a more pronounced conformational change in ICL2 (Fig. 4c). However, direct comparison of these structures is limited because the naloxone-bound inactive MOR structure is stabilized by a negative allosteric modulator (NAM) and the Nb6 nanobody, whereas the morphine-bound MOR represents the active state in complex with Gi. Thus, to further study these conformational changes, we performed unbiased MD simulations in the absence (apo) and presence of naloxone and morphine docked into the inactive MOR structure^23^ (see Methods). Clearly, the carbonyl group of naloxone at the 6-position of the morphinan scaffold (see^41^ for atom notation) forms a more stable hydrogen bond with K235^5.39^ than the hydroxyl group at the corresponding position in morphine **(Fig. S2a)**. The increased stability of this interaction restricts the rotational flexibility of TM5, rendering it more rigid than in the apo state (Figs. 4b and **S2a**) or the morphine-bound state **(Fig. S2a)**. ICL2 of MOR also adopts a conformation distinct from that observed in the apo simulations, resulting in a slight inward shift of ICL2 in D_1_R (Figs. 4c–4d). A conserved Pro residue at position (E/D)R^3.50^YX_5_P, located immediately downstream of the highly conserved (D/E)RY motif, initiates the characteristic two-turn α-helical conformation of ICL2. Consequently, the conformations of ICL2 and the (D/E)RY motif are structurally coupled. Consistent with this rearrangement, the interatomic distance of the ionic interaction between E267^6.30^ in TM6 of D_1_R and R121^3.50^, of the (D/E)RY motif, is reduced (Fig. 4d), indicative of a strengthened ionic interaction and closure of the intracellular cavity required for G protein binding. These findings indicate that naloxone-bound MOR modulates the conformation of ICL2 in D_1_R through the TM4/5 heteromeric interface, thereby influencing its activation process. This hypothesis aligns with the recent structure of active D_1_R bound to the positive allosteric modulator (PAM) LY3154207, which binds near ICL2 and regulates receptor activation (Fig. 4c)^42^. We propose that receptor modulation occurs not only through the classical mechanism, where an allosteric ligand (e.g., LY3154207) binds within the same receptor, but also via ligand binding (e.g., naloxone) to a partner GPCR within a heteromer. In both scenarios, ligand binding induces conformational shifts in ICL2 that modulate receptor activity. To quantify how the local stabilization of K235^5.39^ at the top of TM5 of MOR propagates through the complex to the (D/E)RY motif of D_1_R, we modeled the system as a dynamic communication network **(Fig. S2b-S2e)**.

### Evidence for the existence of functional MOR–D_1_R heteromers in the striatum

Three rigorous criteria have been proposed as necessary to establish the presence of functionally relevant GPCR heteromers in native tissues: first, the heteromer components should colocalize and physically interact; second, heteromers should exhibit properties distinct from those of the protomers (“biochemical fingerprint”); and third, heteromer disruption with reagents or with genetic approaches should lead to a loss of heteromer-specific properties^16^. Several immunochemical techniques have been introduced to provide evidence for GPCR heteromers in native tissue, including proximity ligation assay (PLA) [33]. PLA uses two primary antibodies for the two different GPCRs followed by oligonucleotide-conjugated secondary antibodies to label proteins. When in close proximity (<16 nm), these DNA strands ligate and amplify, appearing as distinct fluorescent puncta that represent a single protein complex. This distance limit is only slightly larger than that of resonance energy transfer (~10 nm), which is a standard method for inferring GPCR oligomerization in transfected cells. This method has for instance been used to detect A_2A_R–D_2_R complexes in rodent and human striatum^44,45^. Positive results with either technique indicate close proximity, suggesting but not demonstrating GPCR heteromerization. Unequivocal demonstration of heteromerization can be obtained by selectively decreasing the number of complexes with heteromer-selective disrupting TM peptides. PLA was then performed in mouse striatal sections as described in the Methods section in the presence and absence of TM peptides of the MOR to detect native MOR–D_1_R heteromers. As shown in Fig. 5a, MOR–D_1_R heteromer complexes could be visualized as red spots. Quantification showed that the PLA signal was significantly reduced by MOR TM4 or TM5 peptides (Figs. 5b, 5c and 5e), which disrupt the transmembrane domains involved in the MOR–D_1_R heteromeric interface. In contrast, the TM6 peptide, used as a negative control because it is not part of the heteromeric interface, had no effect (Figs. 5d and 5e). These results indicate that the detected PLA signal corresponds to true MOR–D_1_R heteromers. Together, these findings fulfill the first criterion by demonstrating the colocalization and physical interaction of both GPCRs.

Radioligand binding experiments have classically shown the existence of two apparent populations of GPCRs, with high and low affinities for the agonist. This has commonly been observed with a shallow or even biphasic curve in radiolabeled antagonist versus agonist competition experiments. Although it has been generally assumed that these results indicate the existence of G protein-coupled and uncoupled states of the GPCR, seminal studies by Redka et al. demonstrated that, within the physiological range of an excess of G proteins versus GPCRs, these apparently different populations of GPCRs correspond to negative binding cooperativity of the agonist binding to the different protomers of GPCR oligomers^46,47^. Application of mathematical models that consider GPCRs as homodimers to the analysis of radioligand binding data, such as the ‘dimer receptor model’, provides values of the two agonist binding affinities (K_DB1_ and K_DB2_) that are significantly more robust (less dependent on the experimental conditions) than those of monomer-G protein models (K_H_ and K_L_)^15,47–50^. The ‘dimer receptor model’ also introduces a dimer cooperativity index (D_C_). D_C_ = 0 implies no agonist cooperativity, whereas positive and negative values imply positive and negative cooperativity, respectively^48^. In a previous study, we used this analysis to evaluate competitive inhibition between [^3^H]-naloxone and endomorphin-1 in HEK-293T cells stably expressing MOR. The ‘dimer receptor model’ showed a significantly better biphasic than monophasic fit, with two affinities and negative cooperativity, demonstrating a predominant population of MOR homodimers^22^. Using the same experimental method in sheep striatal membranes, we also obtained biphasic competitive antagonist curves of [^3^H]-naloxone versus endomorphin-1, with two affinities and negative cooperativity (Fig. 5f). In the presence of the D_1_R agonist SKF38393, but not the D_1_R antagonist SCH23390, the second affinity constant for endomorphin-1 was significantly altered (K_D2_=0.5 μM vs 2.1 μM) (Fig. 5f), indicating that D_1_R activation allosterically modulates biphasic MOR binding. This indirectly demonstrates the presence of a significant population of striatal MORs forming MOR–D_1_R heteromers constituted by MOR homodimers and at least one D_1_R protomer. Competitive antagonist experiments using the D_1_R antagonist [^3^H]-SCH23390 versus the D_1_R agonist SKF38393 were also conducted in sheep striatal membranes. As previously reported, these experiments displayed a biphasic fit with two affinities and negative cooperativity^49^, further supporting the presence of D_1_R homodimers in native striatal tissue (Fig. 5g). However, unlike the effects observed on MOR binding, the MOR ligands endomorphin-1, methadone, and naloxone failed to significantly modify the equilibrium affinity constants of D_1_R for SKF38393 (Fig. 5g). This lack of effect aligns with the absence of MOR-agonist-mediated Type I allosterism on D_1_R agonist-induced G protein coupling or β-arrestin2 recruitment within the MOR–D_1_R heteromer. Furthermore, it implies that Type I allosterism mediated by MOR antagonists does not depend on alterations in D_1_R agonist binding characteristics.

### Biochemical fingerprint of the MOR–D_1_R heteromer in an *in vivo* mouse model

To confirm the MOR–D_1_R heteromer’s biochemical fingerprint in native tissues, we sought to replicate the Type I and III allosterisms found in transfected cells using an *in vivo* mouse model. We monitored intracellular cAMP dynamics in the mouse dorsal striatum using *in vivo* fiber photometry and a genetically encoded biosensor (Figs. 6a–6c). Custom-engineered probes enabled concurrent recording of cAMP-dependent fluorescent signals and microinjection of TM-peptides within the same striatal locus (see Methods and **Fig. S3**). The systemic administration of the D_1_R agonist SKF81297 (0.5–5 mg/kg, i.p.) produced a dose-dependent increase in striatal cAMP formation **(Fig. S4)**. On the hand, methadone (3 mg/kg, i.p.) or naloxone (3 mg/kg, i.p.) did not produce a significant effect (Figs. 6d and 6e), but significantly decreased cAMP formation induced by SKF81297 (1.5 mg/kg, i.p.) (Figs. 6g–6i). These findings demonstrate that two key characteristics of the striatal MOR–D_1_R heteromer, the canonical Gi-Gs antagonistic interaction at AC (Type III allosterism) and cross-antagonism (Type I allosterism), can be identified *in vivo*, translating these biochemical fingerprints from transfected cells to native tissue. Importantly, the third criterion was fulfilled by demonstrating that the heteromer-disrupting TM4 peptide of the MOR, but not the control TM7 peptide, counteracted the naloxone-mediated cross-antagonism of SKF81297 (Figs. 7c–7f). In contrast, control *in vitro* experiments using agarose brain phantom preparations showed no effect for either TM peptide (Figs. 7a and 7b), confirming the specificity of the *in vivo* disruption.

## Discusion

We provide evidence that MOR and D_1_R form functional heteromers both *in vitro* and *in vivo*, with a minimal structural organization consisting of a dimer of homodimers. First, we demonstrate that MOR–D_1_R complexes adopt a heterotetrameric structure where each homodimer is canonically coupled to its respective Gi or Gs protein. This assembly provides the framework for an antagonistic interaction at AC, allowing a MOR agonist to counteract D_1_R-mediated signaling. Second, we show that this heteromer fulfills the three stringent criteria proposed for identifying functionally relevant GPCR heteromers in native tissues. Finally, we propose a structural model that accounts for a key biochemical fingerprint of the complex: a cross-antagonism wherein a MOR antagonist blocks D_1_R signaling.

The potential for striatal MOR–D_1_R heteromerization was previously investigated regarding how D_1_R modulates MOR expression, signaling, and its role in opioid locomotor sensitization in rodents (the progressive increase in locomotion driven by repeated opioid dosing). Thus, repeated morphine administration failed to induce locomotor sensitization in D_1_R knockout mice^51^. That same study demonstrated that the genetic inactivation of D_1_R significantly reduces MOR expression within striatal striosomes^51^. Together, these findings suggested tight, potentially intermolecular interactions between the two receptors. This possibility was initially explored in co-transfected cells using in vitro methods such as co-immunoprecipitation, co-internalization, and BRET assays^17,18^.

However, while these approaches demonstrate close physical proximity, they only confirm that the receptors belong to the same macromolecular complex rather than definitively proving direct heteromerization. To address this limitation, the present study introduces two additional assays: BiLC and CODA-RET. The latter offers a more direct functional readout by measuring G protein activation specifically driven by the heteromeric complex^36^. Ultimately, the most definitive evidence for MOR–D_1_R heteromerization in co-transfected cells was obtained by combining BiLC experiments with specific interfering TM peptides. We subsequently leveraged this strategy using proximity ligation assays (PLA) to demonstrate the presence of the heteromer in the mouse striatum, and to further characterize its biochemical properties.

In the present study, we successfully reproduced the previously reported cross-antagonism wherein a D_1_R antagonist counteracts MOR agonist-mediated signaling. However, we focused our investigation on the potential heteromer-dependent modulatory effects of MOR ligands on D_1_R signaling. Complemented by computational analysis, our results provide further evidence for a common tetrameric architecture, composed of Gs- and Gi-coupled homodimers, as a structural requirement of a macromolecular complex organized as a macromolecular assembly (GEMMA) that mediates canonical Gi-Gs antagonistic interactions at the adenylyl cyclase (AC) level^35^. Furthermore, we identified an opposite cross-antagonism, in which blocking the inactive state of MOR with naloxone counteracts D_1_R signaling within the MOR–D_1_R heteromer. By utilizing TM peptides, we demonstrated that these MOR agonist–D_1_R agonist and MOR antagonist–D_1_R agonist interactions represent novel examples of Type III and Type I allosterism driven by GPCR heteromerization^35^.

Collectively, including our in vivo findings we could demonstrate that MOR–D_1_R heteromers constitute a functionally and pharmacologically significant population of D_1_Rs in the mouse dorsal striatum. This was evidenced by the ability of a systemic D_1_R agonist to drive striatal cAMP formation, which is actively modulated by opioid ligands. Notably, although co-localization of MORs and D_1_Rs is restricted to the striosomal compartment (~20% of the dorsal striatal volume), intrastriatal administration of a selective heteromer-interfering TM peptide significantly counteracted naloxone’s inhibition of a D_1_R agonist-induced cAMP formation. Beyond confirming a dependence on MOR–D_1_R heteromerization, this peptide-specific rescue validates that the systemic compounds alter cAMP signaling through localized striatal receptors. This localized effect is highly consistent with striatal anatomy: the striatum possesses the highest density of D_1_R in the brain, where they are expressed exclusively postsynaptically on one of the two subtypes of GABAergic projection neurons. Consequently, striatal MOR–D_1_R heteromers must reside exclusively within these specific neurons, restricted precisely to the MOR-rich striosomal compartment. This anatomical localization places MOR–D_1_R heteromers in a strategic position to integrate opioid and dopaminergic signaling within striosomal circuits involved in motor control and reward processing.

As reviewed in the Introduction, clinical and experimental evidence supports a key role for striosomal MOR–D_1_R neurons in Restless Legs Syndrome (RLS) and the restlessness of opioid withdrawal. Rodent models of both conditions exhibit an increased sensitivity of these neurons to release GABA. Because both experimental conditions show a reduction in striatal MOR expression without a concomitant decrease in D_1_Rs^12–14^, the dynamic-dependent proportion of MOR–D_1_R heteromers relative to D_1_R homomers should decrease. This reduction in heteromerization would diminish the antagonistic control that MOR exerts over D_1_R signaling within the heteromer, potentially driving the hypersensitivity of striosomal MOR–D_1_R neurons observed in animals and, putatively, in patients. Consequently, striatal MOR–D_1_R heteromers, rather than MORs alone, should be considered the primary target mediating opioid efficacy in RLS. This concept provides a framework for developing safer opioids that preferentially target MOR–D_1_R heteromers over other MOR populations that mediate adverse side effects.

## Methods

### DNA constructs.

For BiLC experiments, human D_1_R and MOR were cloned in-frame into pcDNA3.1 to express amino acid residues 1–229 (nRLuc) or 230–311 (cRLuc) of the RLuc8 protein; non-fused MOR was also used. CODA-RET experiments utilized the following human constructs: Gα_s_-YFP (with the YFP variant mVenus inserted at position 91), untagged Gβ_1_ and Gγ_2_, as well as MOR-nRLuc and D_1_R-cRLuc. For β-arrestin recruitment assays, β-arrestin2-RLuc was used in combination with D_1_R-YFP (Venus variant) and untagged MOR. For NanoBiT experiments, pIRES-HA-PS-GsLgBiT, pIRES-HA-PS-D_1_RSmBiT, and pcDNA3.1-Myc-PS-hMOR constructs were used. All constructs were verified by DNA sequencing.

### TAT-conjugated TM peptides.

A peptide derived from the HIV-transactivator of transcription (TAT; sequence: YGRKKRRQRRR) was fused to peptides corresponding to the transmembrane domains (TM) of human MOR or D_1_R (TM peptides; synthesized by Genemed Synthesis, San Antonio, TX, USA; UPF and CSIC facilities, Barcelona, Spain) to facilitate their integration into the plasma membrane. Because the TAT peptide binds to phosphatidylinositol-(4, 5)-bisphosphate localized on the inner leaflet of the plasma membrane^21^, TAT was fused to the N-terminus of TM2, TM4, and TM6 peptides, and to the C-terminus of TM1, TM3, TM5, and TM7 peptides. This design was chosen to ensure the correct orientation of each inserted TM domain. The amino acid sequences of these constructs were as follows:

D_1_R-TM4 YGRKKRRQRRRAAFILISVAWTLSVLISFIPVQLSW
D_1_R -TM5 TYAISSSLISFYIPVAIMIVTYTSIYYGRKKRRQRRR
D_1_R -TM6 YGRKKRRQRRRTLSVIMGVFVCCWLPFFISNCMVPFCG
D_1_R -TM7 FDVFVWFGWANSSLNPIIYAFNADFYGRKKRRQRRR
MOR-TM1 TAITIMALYSIVCVVGLFGNFLVMYYGRKKRRQRRR
MOR-TM2 YGRKKRRQRRRIYIFNLALADALATSTLPFQSVNYL
MOR-TM3 KIVISIDYYNMFTSIFTLCTMSVYGRKKRRQRRR
MOR-TM4 YGRKKRRQRRRAKIINVCNWILSSAIGLPVMFM
MOR-TM5 ENLLKICVFIFAFIMPVLIITVCYGYGRKKRRQRRR
MOR-TM6 YGRKKRRQRRRITRMVLVVVAVFIVCWTPIHIYVIIKA
MOR-TM7 FQTVSWHFCIALGYTNSCLNPVLYYGRKKRRQRRR

### Cell culture and stable cell line generation.

Human embryonic kidney 293T (HEK-293T) cells were obtained from the ATCC and maintained below passage 20. HEK-293T cells stably expressing the MOR were generated as previously described^52^. To generate the double-stable MOR–D_1_R cell line, human D_1_R cDNA (Missouri S&T cDNA Resource Center) was modified at the N-terminus with an in-frame signal peptide to enhance cell-surface expression, followed by a Myc epitope tag, and subcloned into pcDNA3.1. The construct was verified by DNA sequencing. This plasmid was then transfected into MOR cells, and stable clones were selected using hygromycin B (50 μg/mL) and Geneticin (G-418, 400 μg/mL). Cells were cultured in Dulbecco’s modified Eagle’s medium (DMEM; Merck KGaA, Darmstadt, Germany) supplemented with 2 mM L-glutamine, 100 U/mL penicillin/streptomycin, MEM non-essential amino acid solution (1/100), and 5% (v/v) heat-inactivated fetal bovine serum (FBS) (all supplements from Gibco, Thermo Fisher Scientific, MA, USA) in a humidified incubator at 37°C with 5% CO_2_. G-418 (400 μg/mL; Biowest, Nuaillé, France), and hygromycin B (50 μg/mL; Corning, NY, USA) were also added in the culture medium for the stable cell lines. For NanoBiT experiments, HEK-293T cells were cultured in DMEM, prewarmed to 37 °C and supplemented with 5% (v/v) FBS, 75 U/mL penicillin, 75 μg/mL streptomycin, and 2 mM L-glutamine (all supplements from Gibco).

### Bimolecular luminescence complementation (BiLC) Assay.

HEK-293T cells seeded in 6-well plates were co-transfected using polyethylenimine (PEI). Cells were transfected with cDNAs encoding the target receptors fused to complementary RLuc8 fragments (1.5 μg of each plasmid). To analyze receptor heteromerization, cells were co-transfected with MOR-nRLuc and D_1_R-cRLuc. To analyze receptor D_1_R homomerization, cells were co-transfected with D_1_R-nRLuc and D_1_R-cRLuc in the presence and absence of non-fused MOR. Forty-eight hours post-transfection, cells were treated with or without the indicated TAT-TM peptides (2 μM) for 4 h at 37 °C. Reconstituted RLuc8 luminescence was quantified at 485 nm using a Mithras LB940 microplate reader, 10 min after the addition of coelenterazine H.

### cAMP formation assay.

Homogeneous time-resolved fluorescence energy transfer (HTRF) assays were performed using the Lance Ultra cAMP kit (PerkinElmer, Shelton, CT, USA), which is based on the competitive displacement of a europium chelate-labeled cAMP tracer bound to a specific antibody conjugated to acceptor beads. HEK-293T cells stably expressing MOR and D_1_R were utilized. Two hours prior to the experiment, the culture medium in the 25-cm^2^ cell flasks was replaced with non-supplemented medium. Subsequently, cells were detached, resuspended in medium containing 30 μM zardaverine, and counted. Cells were then seeded at a density of 1,200 cells per well in ProxiPlate 384-well microplates (PerkinElmer). After seeding, cells were treated at 25°C for 10 min with either the D_1_R antagonist SCH23390, the MOR antagonist naloxone, or the corresponding vehicle. Cells were then stimulated with the MOR agonist endomorphin-1, the D_1_R agonist SKF38393, or their respective vehicles for 15 min. Following this, 0.5 μM forskolin or vehicle was added, and the mixture was incubated for an additional 15 min. In some experiments, cells were pretreated with or without the indicated TAT-TM peptides (2 μM) for 4 h at 37 °C before antagonist or agonist treatment. Finally, HTRF fluorescence emission at 665 nm was analyzed using a PHERAstar Flagship microplate reader equipped with an HTRF optical module (BMG LABTECH, Offenburg, Germany).

### NanoBiT assay.

HEK-293T cells were transiently cotransfected with Gs-LgBiT, D_1_R-SmBiT and non-fused MOR using branched 25 kDa PEI at a 1:3 DNA-to-PEI mass ratio (w/w). Four hours post-transfection, the culture medium was replaced with fresh DMEM, and cells were cultured at 37 °C in 5% CO_2_ for 24 h. Transfected cells were then re-plated at a density of 50,000 cells/well into poly-D-lysine-coated white 96-well optical-bottom microplates (Nunc^™^, Thermo Fisher Scientific, Waltham, MA, USA). After 24 h, drugs prepared in Dulbecco’s Phosphate Buffered Saline (DPBS; Gibco) were added. Cells were pretreated with vehicle, naloxone hydrochloride (Sigma-Aldrich, St. Louis, MO, USA), or methadone (NIDA Pharmacy, Baltimore, MD, USA) for 10 min, followed by a 10 min incubation with SKF81297 hydrobromide (Tocris Bioscience, Bristol, UK). Following treatment, cells were washed once with DPBS and maintained in 90 μL of DPBS. Next, 10 μL of 5 μM h-coelenterazine (NanoLight Technologies, Pinetop, AZ, USA) was added. After a 5 min room-temperature incubation, luminescence was recorded using a Mithras LB 940 multimode reader (Berthold Technologies, Bad Wildbad, Germany) and expressed as integrated relative light units (RLU).

### Complemented donor-acceptor resonance energy transfer (CODA-RET) and β-arrestin2 recruitment assays.

For the CODA-RET assay, HEK-293T cells were co-transfected with 0.9 μg of D_1_R-nRLuc8 and 3.6 μg of MOR-cRLuc8), Gαs-mVenus (5 μg), untagged Gβ_1_ (4.5 μg) and Gγ_2_ (5 μg). For β-arrestin2 recruitment assays, HEK-293T cells were co-transfected with 9 μg of D_1_R-mVenus and 10 μg of β-arrestin2-RLuc. Transfections were performed using PEI in 100-cm^2^ cell culture dishes. Four hours post-transfection, the culture medium was replaced with fresh DMEM, and cells were cultured at 37 °C in 5% CO_2_ for 24 h. All experiments were carried out approximately 48-hours after transfection. Cells were harvested, washed, and resuspended in HBSS. Approximately 200,000 cells/well were distributed in 96-well plates, and 5 μM coelenterazine H was added to each well. Agonists were added 2.5 minutes after coelenterazine H. When indicated, agonists or antagonists of the partner receptor were added 10 minutes prior to coelenterazine H to assess the modulation of the partner’s receptor signaling. The fluorescence of the acceptor (mVenus variant of YFP) was measured (excitation at 405 nm and emission at 535 nm) in a Mithras LB940 to confirm the constant expression levels across experiments. In parallel, BRET signals were determined from the same batch of cells as the ratio of the light emitted by mVenus (535 nm) to that emitted by RLuc (485 nm). G protein-activation and β-arrestin2 recruitment were calculated as the increase in BRET ratio (mVenus/RLuc) for each drug concentration minus the BRET ratio in the absence of ligand, measured 10 minutes after ligand addition.

### Animals.

Male and female C57Bl/6J mice (Charles River Laboratories, Wilmington, MA) were used for fiber photometry experiments and male C57Bl/6J mice (Animal Service, University of Barcelona, Barcelona, Spain) were for used for the PLA experiments. Mice were housed (four per cage) and kept on a 12-hour light/12-hour dark cycle with food and water available *ad libitum*. All animals used in the study were maintained in accordance with the guidelines of the National Institutes of Health (NIH) Animal Care, and the animal research conducted to perform this study was reviewed and approved by the NIDA IRP Animal Care and Use Committee (protocol #24-MTMD-27). Procedures using rodents were also approved by the Ethical Committee for Animal Use of the University of Barcelona (OB 408/18 and OB 409/18).

### In situ proximity ligation assay (PLA).

Mice were sacrificed by cervical dislocation, and their brains were rapidly harvested and immersed in ice-cold, oxygenated (95% O_2_ / 5% CO_2_) Krebs-HCO3^−^ buffer (in mM: 124 NaCl, 4 KCl, 1.25 KH_2_PO_4_, 1.5 MgCl_2_, 1.5 CaCl_2_, 10 glucose, and 26 NaHCO_3_, pH 7.4). Coronal slices (500 μm thick) were cut at 4°C in the same buffer, gradually warmed to 30°C, and incubated under constant oxygenation (95% O_2_ / 5% CO_2_) for 4–5 h in an Eppendorf Thermomixer (5 Prime, Boulder, CO, USA) with or without TM peptides (4 μM). Following incubation, slices were fixed in 4% paraformaldehyde (1 h at 4°C), washed in Tris-buffered saline (50 mM Tris-HCl, 0.9% NaCl, pH 7.8), cryoprotected in 30% sucrose (24 h at 4°C), and stored at −20°C. Coronal sections (20 μm thick) were subsequently cut on a freezing cryostat (Leica Jung CM-3000), mounted on glass slides, and frozen at −20°C until further use. Receptor complexes were detected using the Duolink *in situ* PLA Detection Kit (Sigma-Aldrich) according to the manufacturer’s protocol. Sections were thawed, washed in PBS, and permeabilized with 0.01% Triton X-100 in PBS for 10 min. MOR–D_1_R complexes were labeled using a mixture of mouse anti-MOR monoclonal antibody (1:200; Cat# 677014, Invitrogen) and guinea pig anti-D_1_R antibody (1:200; Cat# D1R-GP-Af500, Frontier Institute). Secondary detection was performed using anti-mouse MINUS (1:5) and custom-conjugated anti-guinea pig PLUS (1:20; conjugated via Duolink kit) PLA probes. Slides were mounted with DAPI-containing mounting medium and imaged on a Zeiss LSM880 confocal microscope using a 63X oil-immersion objective (N.A. 1.4). Z-stacks (9–15 optical sections at 1 μm intervals) were acquired using 405 nm and 561 nm laser lines. Images were processed using custom Python scripts (aicspylibczi, scipy, and scikit-image packages). Following maximum-intensity z-projection of .czi files, nuclei were segmented from the blue channel via adaptive histogram equalization, Gaussian filtering, and Otsu thresholding, with watershed segmentation applied to separate individual nuclei. Red channel puncta were detected using a global intensity threshold derived from negative controls, followed by Gaussian smoothing, and watershed segmentation was used to separate merged dots. Puncta were assigned to cells by expanding nuclear masks by a fixed radius; those falling outside were excluded. Cells containing at least one punctum were classified as positive cells. Quantification was expressed as the proportion of positive cells relative to total nuclei. A total of 1,500–3,000 cells from 12–20 fields (63X) for each condition were analyzed across five animals. RGB composite overlays were generated for quality control.

### In vivo fiber photometry.

Anesthetized mice (ketamine/xylazine, 60–100/5–15 mg/kg, i.p.) were placed in a stereotaxic frame. An adeno-associated virus encoding the cAMP biosensor cADDis (pAAV2-hSyn Green Upward cADDis, Montana Molecular; 0.5 μL, 2.7 × 10^12^ GC/mL) was injected unilaterally into the dorsal striatum (AP −1.0 mm, ML +1.5 mm, DV −3.0 mm from bregma) at 0.05 μL/min. The needle remained in place for 10 min post-infusion. A chronic fiber-optic implant (200 μm core, 0.50 NA; Thorlabs, Newton, NJ) or a custom photo-fluidic probe **(Fig. S2)** was placed at the same coordinates and secured with screws and dental cement. Recordings were conducted using a dual-wavelength fiber-photometry system (405 nm isosbestic, 465 nm biosensor; emission 500–550 nm; LUX RZ10X processor, Tucker-Davis Technologies, Alachua, FL, USA). Patch cords were photobleached for 2 h prior to use. Mice were acclimated in rotating chambers, with the initial 20 min excluded for stabilization. In the first experiment, A 20-min baseline was recorded before systemic administration of methadone HCl (3 mg/kg), naloxone (3 mg/kg), or saline. In the second experiment, following a 20-min baseline, mice received systemic methadone HCl (3 mg/kg), naloxone (3 mg/kg), or saline, followed 15 min later by SKF81297 hydrobromide (1.5 mg/kg, i.p.). For the experiments using photo-fluidic probes, TM4 or TM7 (50 μM) in artificial cerebrospinal fluid (144 mM NaCl, 4.8 mM KCl, 1.7 mM CaCl_2_, and 1.2 mM MgCl_2,_ 0.1% DMSO) was microinfused into the dorsal striatum at 5.0 nL/min for 100 min. At 40 min post-infusion, mice received naloxone (3.0 mg/kg, i.p.), followed 15 min later by SKF81297 hydrobromide (1.5 mg/kg, i.p.). Fluorescence was recorded continuously post-injection. Signals were acquired using Synapse software and raw data were extracted via pMAT (v1.223). Analysis was performed in MATLAB R2023b (MathWorks, Natick, MA, USA). Raw 405-nm and 465-nm signals were smoothed using locally weighted scatterplot smoothing (LOWESS; span = 0.002). To correct for motion artifacts and photobleaching, the baseline relationship between signals was estimated via linear regression. The fitted 405-nm reference signal was applied across the entire recording. Normalized fluorescence (ΔF/F) was calculated as (*F*_465_ – *F*_405,fit_)/*F*_405,fit_ × 100, where *F*_405,fit_ represents the fitted 405 nm reference signal. Individual traces were aligned to drug administration (*t* = 0), binned into 10-s intervals, and averaged to calculate group mean ± SEM. For sequential TM peptide, naloxone, and SKF81297 experiments, the area under the curve (AUC) for ΔF/F was calculated over 0–20 min post-SKF81297 administration. At the end of the experiments, mice were deeply anesthetized and transcardially perfused with PBS followed by 4% formaldehyde. Brains were processed as previously described^12^. Probe placement and cADDis expression in the dorsal striatum were verified in 40-μm coronal sections using a Typhoon multimode laser scanner (GE Life Sciences, Piscataway, NJ, USA).

### Radioligand binding experiments.

Radioligand binding assays were performed on striatal tissue from 4–6-month-old sheep, freshly obtained from a local slaughterhouse. Tissues were homogenized (3 × 5 s; Polytron PTA 7 rotor, setting 3; Kinematica) in 10 volumes of 50 mM Tris-HCl (pH 7.4) with protease inhibitors (Sigma-Aldrich). Membranes were isolated by two rounds of centrifugation (105,000 × *g*, 45 min, 4°C). Pellets were stored at −80°C, washed once, and resuspended in Tris-HCl buffer. Protein concentration was quantified via BCA assay (Pierce) against BSA standards. Assays were carried out using membrane suspensions (0.3 mg protein/mL) in 50 mM Tris-HCl (pH 7.4) with 5 mM MgCl_2_ at room temperature for 2 h. For MOR competition assays, membranes were incubated with 1.2 nM [^3^H]-naloxone, increasing endomorphin-1 concentrations, and 100 nM of either SCH23390 (D_1_R antagonist) or SKF38393 (D_1_R agonist). For D_1_R competition assays, membranes were incubated with 0.85 nM [^3^H]-SCH23390, increasing SKF38393 concentrations, and 100 nM of MOR ligands (endomorphin-1, methadone, or naloxone). Bound ligands were isolated by rapid filtration of 400 μL aliquots through 0.3% PEI-treated Whatman GF/C filters using a Brandel cell harvester, followed by a 5 s wash with 4.5 mL ice-cold Tris-HCl buffer. Filters were incubated overnight in 5 mL Ultima Gold MV cocktail (PerkinElmer), and radioactivity was measured on a Tri-Carb 2900 scintillation counter (62% efficiency). Data were analyzed by nonlinear regression using the commercial Grafit curve-fitting software (Erithacus Software, Surrey, UK), by fitting the binding data according to the ‘dimer receptor model’^48^, which provides the macroscopic equilibrium dissociation constants describing the binding of the first and second ligand molecules to the receptor homodimer, K_D1_ and K_D2_, respectively. K_DAB_ represents the affinity of the competing ligand for the second protomer when the first protomer is already occupied by the radioligand.

### Computational methods.

Computational models of the MOR–MOR and D_1_R–D_1_R homodimers were created using the TM5/6 dimerization interface of the MOR structure (PDB code 4DKL)^23^, and the MOR–D_1_R heterodimer was modeled based on the TM4/5 interface identified in the ß_1_-adrenergic receptor (4GPO)^53^. The structures of inactive MOR (4DKL), active MOR in complex with Gi (8EF6)^54^, active D_1_R in complex with Gs (7JOZ)^55^ and the previously obtained homology model of inactive D_1_R^56^ were used. Three heterotetrameric models were constructed to investigate alternative G protein arrangements **(Fig. S1)**. Cross-antagonism was investigated using computational models of the MOR–D_1_R heterodimer comprising inactive D_1_R in the apo state and MOR in the inactive apo, naloxone-bound, or morphine-bound states. Naloxone- and morphine-bound MOR models were generated by docking the respective ligands into the inactive MOR conformation (4DKL), using the MOR-naloxone (9BJK)^57^ and MOR-morphine (8EF6)^54^ cryo-EM structures as template-guided docking references. These initial systems were prepared for MD simulation using AmberTools24^58^ and embedded in a rectangular lipid bilayer box, constructed using PACKMOL-memgen^59^, containing 1-palmitoyil-2-oleoyl-sn-glycero-3-posphocholine (POPC), water molecules and a 0.15 M concentration of monoatomic Na+ and Cl- ions. MD simulations of these systems were performed with GROMACS using the amber14sb-ildn forcefield for the protein, solvent and ions^60^, lipid14 for the lipids^61^, and the general Amber force field (GAFF2) with HF/6–31G*-derived RESP atomic charges for the ligands, using the protocol previously reported^62^. The analysis of the trajectories was performed using MDAnalysis^63^, cpptraj^58^, and GetContacts (https://getcontacts.github.io/).

## Supplementary Material

Supplementary Files

This is a list of supplementary files associated with this preprint. Click to download.

- SupplementaryInformation.docx

## Figures and Tables

**Figure 1.**
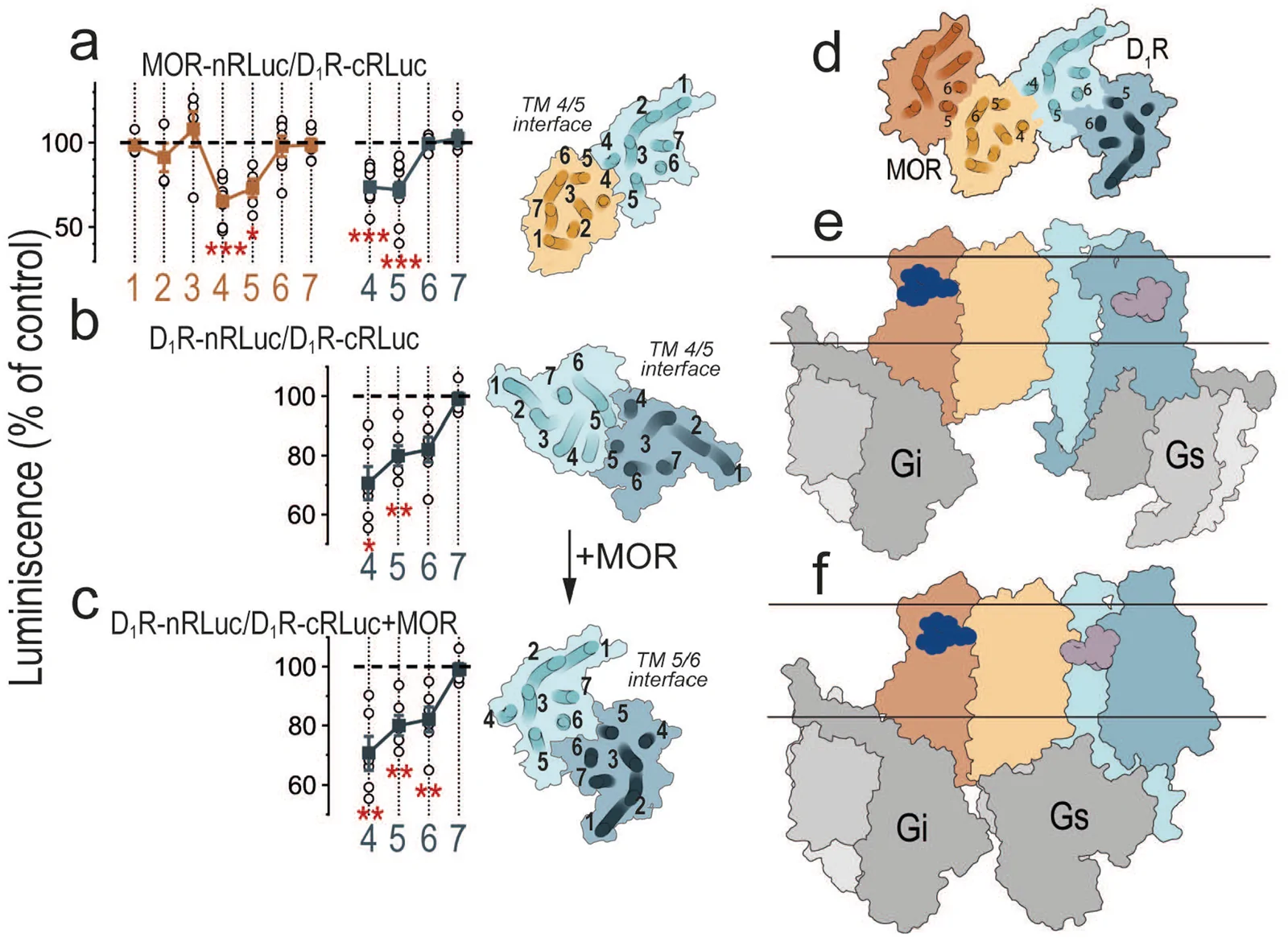
Identification of transmembrane domains mediating MOR–D_1_R heteromerization and D_1_R-D_1_R homomerization. Bimolecular luminescence complementation (BiLC) assays showing interactions between (**a**) MOR-nRLuc and D_1_R-cRLuc, (**b**) D_1_R-nRLuc and D_1_R-cRLuc in the absence of MOR, and (**c**) D_1_R-nRLuc and D_1_R-cRLuc in the presence of MOR. Luminescence resulting from RLuc complementation was quantified in HEK-293 cells in the absence or presence of MOR-derived TM1–TM7 peptides **(a)** and D_1_R-derived TM4–TM7 peptides (**a**-**c**). Cells were treated for 4 h with vehicle or the corresponding TM peptide (2 μM) prior to the complementation assay. The schemes illustrate the corresponding interfaces. ata were normalized to the vehicle control (100%) for each independent experiment and are expressed as mean ± SEM from 4–13 independent experiments, each performed in triplicate. Basal luminescence values were (1.95 ± 0.14) × 10^5^ arbitrary units for **a**, (7.0 ± 0.5) × 10^5^ arbitrary units for **b** and (5.8 ± 0.7) × 10^5^ arbitrary units for **c**. Normality was assessed using the Shapiro–Wilk test. Statistical significance was evaluated using a one-sample, one-tailed t-test against the control value (100%). P values were adjusted for multiple comparisons using the Holm–Bonferroni procedure. Adjusted P values of < 0.05 (*), < 0.01 (**), and < 0.001 (***) indicate significantly lower luminescence values than the control. (**d**) Computational model of the MOR–D_1_R heterotetramer built using the experimental interfaces predicted in panels **a**–**c** (TM4/5 for heterodimerization and TM5/6 for homodimerization). (**e**, **f**) Schematic representation of the MOR–D_1_R heterotetramer viewed parallel to the membrane. (**e**) Model in which the outer protomer of the MOR homodimer is coupled to Gi, whereas the outer protomer of the D_1_R homodimer is coupled to Gs. (**f**) Alternative model in which the outer protomer of the MOR homodimer is coupled to Gi, whereas the inner protomer of the D_1_R homodimer is coupled to Gs.

**Figure 2.**
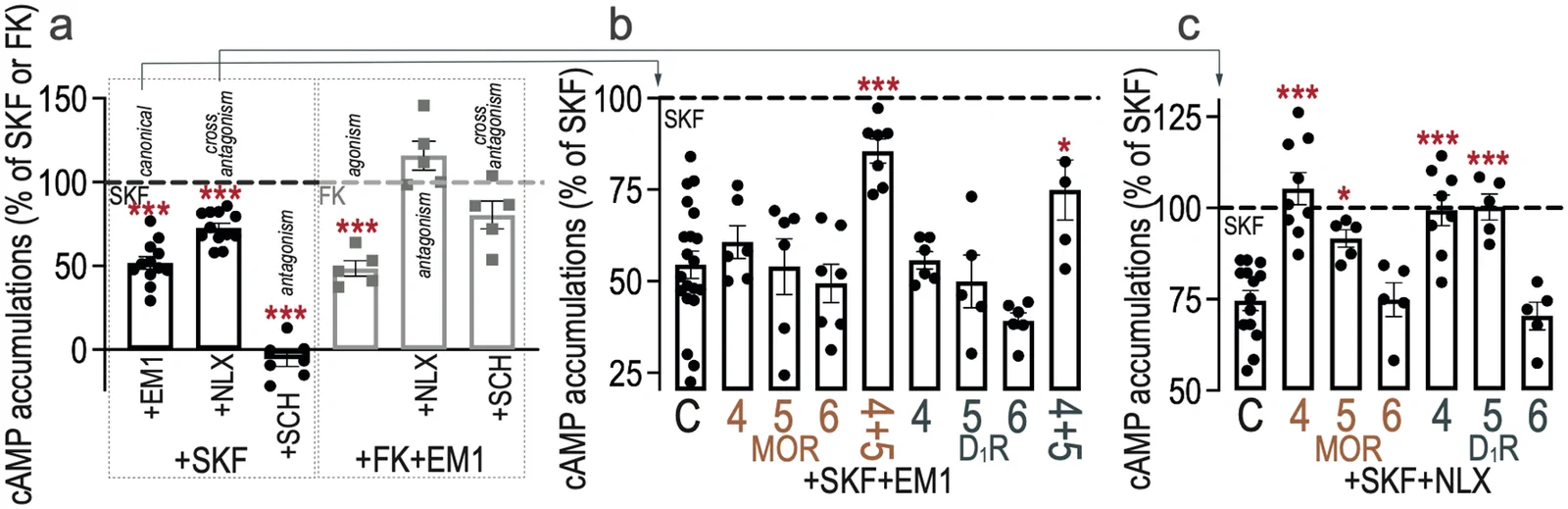
Canonical Gi-Gs antagonistic interaction and cross-antagonism between MOR and D_1_R in cAMP formation experiments. (**a**) cAMP accumulation assays were performed in stably transfected MOR–D_1_R HEK-293T cells. Cells were pretreated for 10 min with the MOR antagonist naloxone (NLX; 1 μM), the D_1_R antagonist SCH23390 (SCH; 1 μM) or vehicle, followed by stimulation for 15 min with the MOR agonist endomorphin-1 (EM1; 1 nM) or the D_1_R agonist SKF38393 (SKF; 1 μM), and an additional 15 min with forskolin (FK; 0.5 μM). Data are expressed as mean ± SEM from 5–14 independent experiments performed in quadruplicate, normalized to the FK- or SKF-stimulated response (100%) for each experiment. Baseline 100% values were 150 ± 55 pmol/million cells (FK) and 110 ± 30 pmol/million cells (SKF). Statistical significance was evaluated using a one-sample t-test comparing each condition to the SKF or FK reference, which was normalized to 100% (broken line). P values were corrected for multiple comparisons using the Holm–Bonferroni procedure. Adjusted P< 0.001 is denoted by ***. Effects of disruptive TM peptides on (**b**) canonical Gi-Gs antagonistic interaction and (**c**) cross-antagonism assessed by cAMP accumulation assays in MOR–D_1_R HEK-293T cells. cAMP accumulation was measured in MOR–D_1_R cells preincubated for 4 h with TM4 (4), TM5 (5), or TM6 (6) peptides (2 μM) from MOR (in brown) or D_1_R (in dark blue). Cells were subsequently treated for 15 min with EM1 (1 nM) or NLX (1 μM), followed by stimulation for an additional 15 min with SKF38393 (SKF; 1 μM). Values are expressed as percentage of SKF response (150 ± 20 pmol/million cells) and represent means ± SEM from 4–18 independent experiments performed in quadruplicate. Normality was assessed using the Shapiro–Wilk test. Comparisons versus control (C, no peptide) were analyzed using one-way ANOVA followed by Dunnett’s multiple comparisons test (*p < 0.05, ***p <0.001).

**Figure 3.**
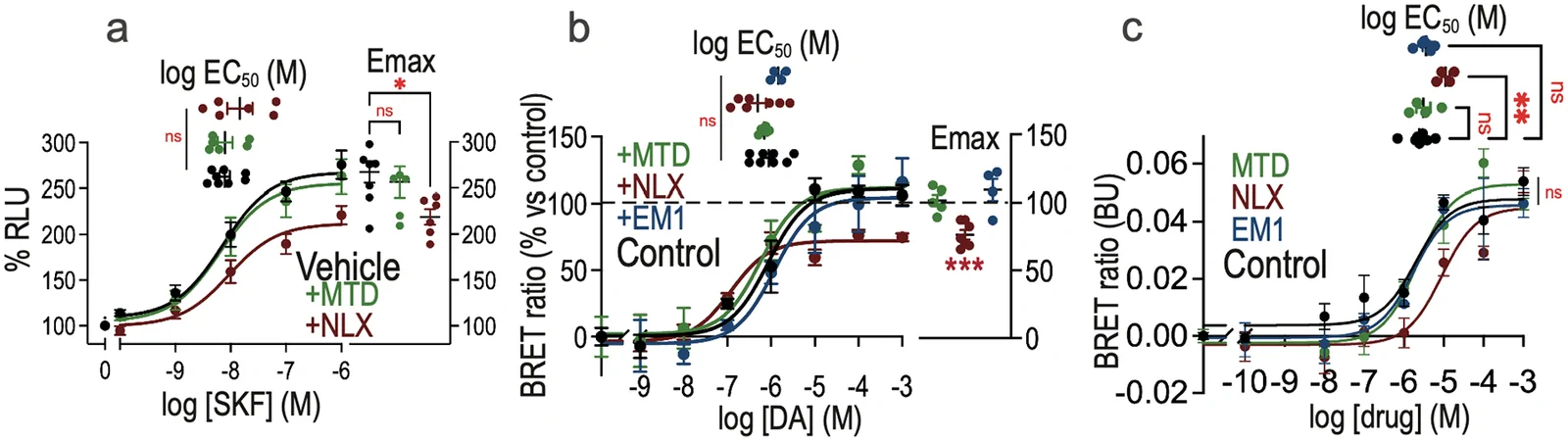
Cross-antagonism between MOR and D_1_R in G protein coupling and β-arrestin recruitment experiments. (**a**) NanoBiT experiments in HEK-293T cells cotransfected with D_1_R-SmBiT and Gs-LgBiT and non-fused MOR. Concentration−response curves showing SKF81297 (SKF)-induced increase in coelenterazine-driven NanoLuc luciferase bioluminescence, in the absence (vehicle) and presence of methadone (+MTD) and naloxone (+NLX). Results were normalized to the Relative Luminescence Units (RLU) produced in the absence of SKF (0%). E_max_ and log EC_50_ of the NanoBiT assay (mean ± SEM; n = 8 in all experiments, with triplicates) are shown. Comparisons of Emax and log EC_50_ versus vehicle were analyzed using one-way ANOVA followed by Dunnett’s multiple comparisons test (*p < 0.05). (**b**) CODA-RET experiments were performed in HEK-293T cells co-transfected with MOR-cRLuc, D_1_R-nRLuc and Gαs-YFP. Concentration-response curves of dopamine (DA)-induced changes in BRET signal were obtained in the absence (Control) or presence of EM1 or MTD (1 μM) or NLX (10 μM). E_max_ and log EC_50_ of the CODA-RET experiments (mean ± SEM from 4–8 independent experiments performed in triplicate) are shown. Statistical significance was evaluated using a one-sample t-test comparing each condition to the maximal BRET ratio produced by dopamine (DA, 100%, broken line). P values were corrected for multiple comparisons using the Holm–Bonferroni procedure. Adjusted P< 0.001 is denoted by ***. (**c**) β-arrestin recruitment experiments were performed in HEK-293T cells co-transfected with β-arrestin2-Rluc, D_1_R-YFP, and non-fused MOR were stimulated with increasing concentrations of DA in the absence or presence of fixed concentrations (10 μM) of NLX, EM1, or MTD. β-arrestin recruitment curves are expressed as BRET units (BU) and data represent the mean ± SEM of one representative experiment performed in triplicate. LogEC_50_ of the β-arrestin experiments (mean ± SEM from 4–6 independent experiments performed in triplicate) are shown. Statistical significance was determined using one-way ANOVA followed by Dunnett’s multiple comparisons *post hoc* test. P< 0.01 is denoted by **.

**Figure 4.**
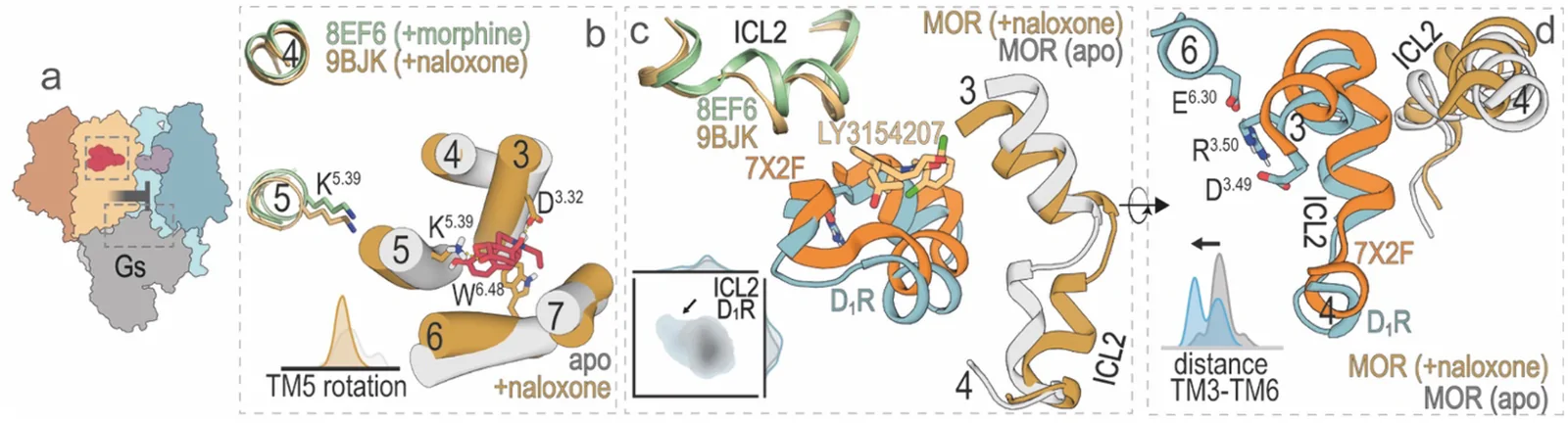
Mechanism by which naloxone-bound MOR induces cross-antagonism of the partner D_1_R. (**a**) Naloxone (in red) binds the inward-facing, inactive, Gi-unbound protomer of the MOR homodimer, whereas SKF38393 (in gray) binds the inward-facing, active, Gs-bound protomer of the D_1_R homodimer. (**b**) Structural comparison of the extracellular ends of TM4 and TM5 in the naloxone-bound (PDB: 9BJK) and morphine-bound (PDB: 8EF6) MOR cryo-EM structures (left). Three independent 1-μs unbiased MD simulation replicas of the MOR–D_1_R heterodimer in the apo state of MOR and naloxone-bound MOR were performed (right). The hydrogen bond between K235^5.39^ and the carbonyl group of naloxone, restricts the rotational flexibility of TM5 (inset). (**c**) Structural comparison of ICL2 in the naloxone-bound and morphine-bound MOR cryo-EM structures (top). A representative conformation of ICL2 of the apo state of MOR (gray), naloxone-bound MOR (brown), and D_1_R (light blue) obtained during the MD simulations of the MOR–D_1_R heterodimer (bottom). Contour plots (inset) of the evolution of the center of mass of amino acids forming ICL2 of D_1_R during three replicas of unbiased 1-μs MD simulations of the MOR–D_1_R heterodimer in the apo state of MOR and naloxone-bound MOR. Distributions of the x and y values are shown on the top x-axis and right y-axis, respectively. The cryo-EM structure of active D_1_R (orange) in complex with the PAM LY3154207 (light orange) is superimposed on the representative D_1_R structure obtained from the simulations (light blue). (**d**) Rotated view of the structures shown in panel **c**. The movement of ICL2 modifies the conformation of the highly conserved (D/E)RY motif, reducing the interatomic distance of the ionic interaction between R121^3.50^ in TM3 and E267^6.30^ in TM 6 and R121^3.50^ (inset).

**Figure 5.**
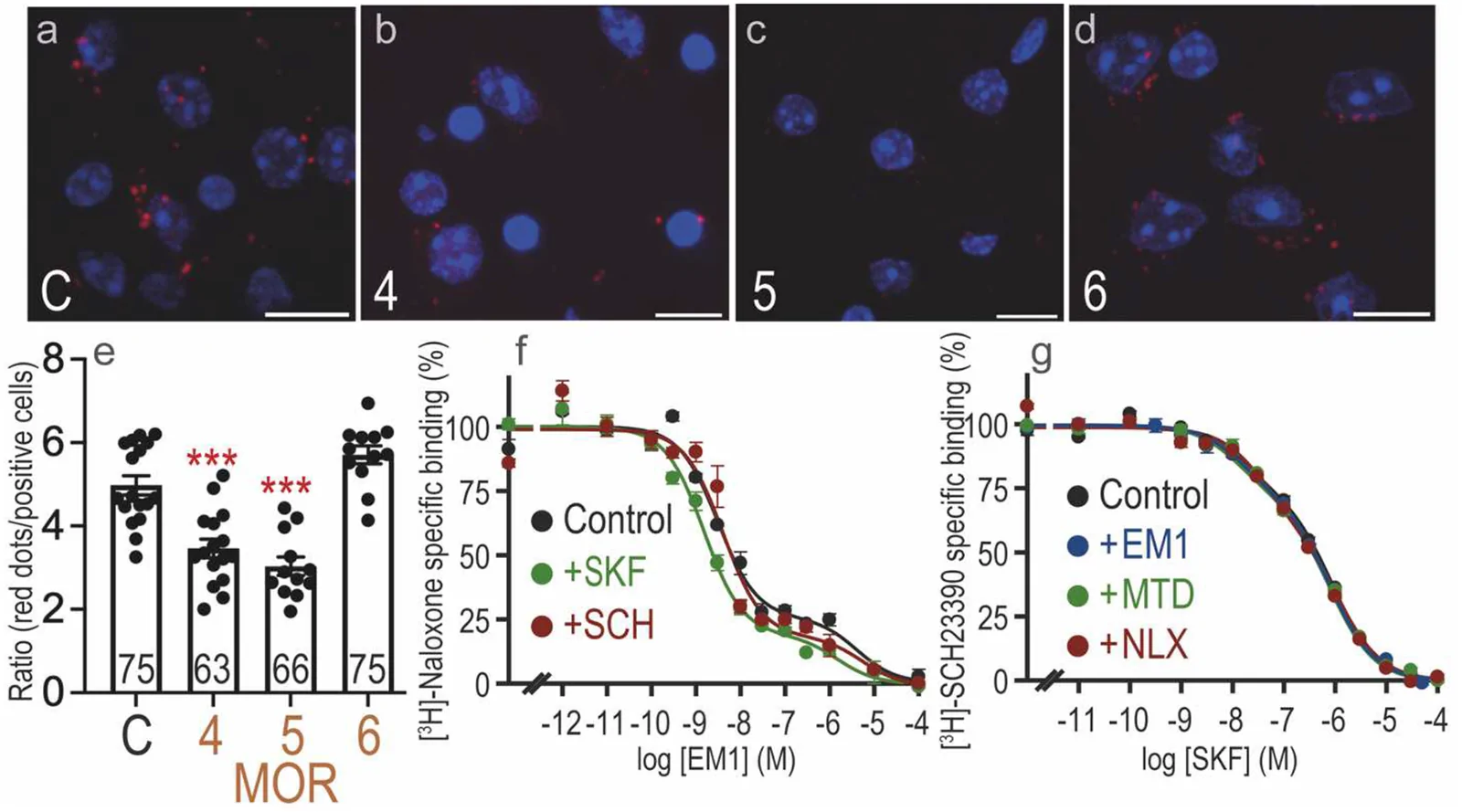
Detection of MOR–D_1_R heteromers in mouse striatum and disruption by interface-interfering TM peptides. (**a**-**d**) In situ proximity ligation assays (PLA) were performed in mouse striatal sections as described in the Methods section to detect endogenous MOR–D_1_R complexes. Representative confocal microscopy images of control samples treated with vehicle (C, **a**), the interface-disrupting TM4 (4, **b**) and TM5 (5, **c**) peptides of MOR, or the negative control TM6 (6, **d**) peptide of MOR are shown. MOR–D_1_R heteromers are visualized as red spots, whereas cell nuclei were counterstained with DAPI (blue). Scale bar represents 10 μm. (**e**) Quantification of the PLA signal, expressed as the number of red dots per positive cell. Values represent means ± SEM of counts from 12–20 different fields from each condition from 5 different animals. Comparisons versus control were analyzed using one-way ANOVA followed by Dunnett’s multiple comparisons test ***P< 0.001 vs. control). The percentage of positive cells is indicated within each bar. **f**-**g**) Competitive radioligand binding experiments in sheep striatal membrane preparations. (**f**) Representative competition curves of [^3^H]-naloxone with increasing concentrations of the MOR agonist endomorphin-1 (EM1) in the absence (black curve) or presence of a constant concentration of 100 nM of the D_1_R agonist SKF38393 (SKF, green curve), or the D_1_R antagonist SCH23390 (SCH, red curve). (**g**) Representative competition curves of [^3^H]-SCH23390 with increasing concentrations of SKF in the absence (black curve) or presence of a fixed concentration (100 nM) of EM1 (blue curve), methadone (MTD, green curve), or the MOR antagonist naloxone (NLX, red curve). Data were fitted using the dimer receptor model^48^. Values represent means ± SEM from one representative experiment out of four independent experiments performed in triplicate (see Table S1 for affinity constants and statistical analyses). 100% corresponds to 0.14 ± 0.02 (**f**) or 0.5 ± 0.1 (**g**) pmol/mg of protein.

**Figure 6.**
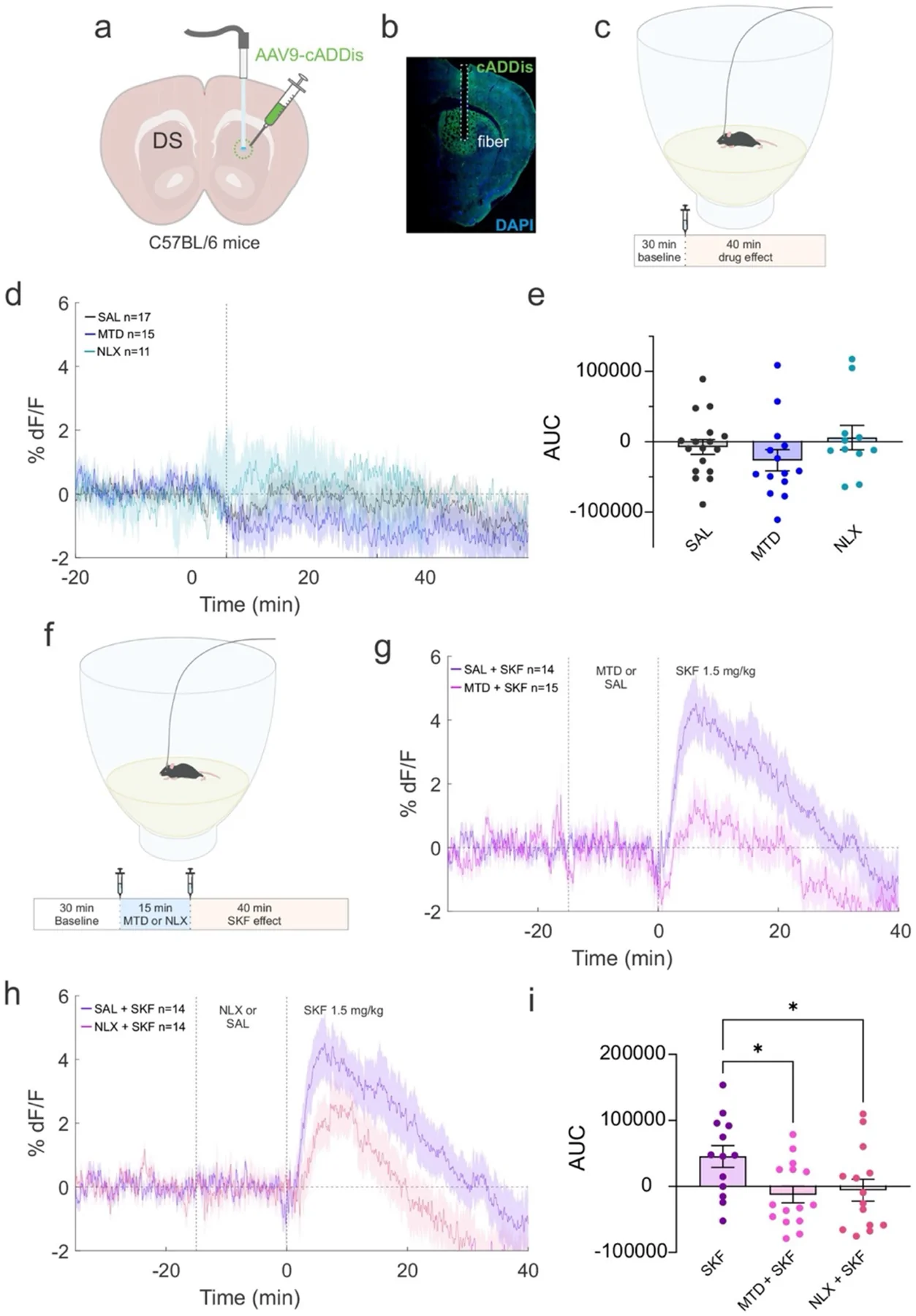
*In vivo* antagonistic interaction between MOR and D_1_R in the modulation of neuronal cAMP in the mouse dorsal striatum. (**a**) Schematic representation of the strategy used to express the cADDis biosensor in the striatum. (**b**) Representative histological image showing cADDis expression in the mouse dorsal striatum. (**c**) Experimental fiber photometry strategy. (**d**) Effect of systemic administration of methadone (MTD; 3.0 mg/kg), naloxone (NLX; 3.0 mg/kg), or saline (SAL) on striatal cAMP levels. (**e**) Area under the curve (AUC) measured following administration of MTD, NLX, or SAL. (**f**) Experimental fiber photometry strategy. Attenuating effect of methadone (**g**) and naloxone (**h**) on SKF81297 (SKF; 1.5 mg/kg)-induced increase in striatal cAMP. (**i**) AUC measured following administration of SKF after a 15-min pretreatment with MTD or NLX. Shaded areas (**d**, **g**, and **h**) and error bars (**e** and **i**) represent SEM. Vertical dotted lines indicate the time of systemic administration. Horizontal dotted lines indicate baseline fluorescence (set to 0%). Comparisons to the control (SAL) group were analyzed using one-way ANOVA followed by Dunnett’s multiple-comparisons test. *P< 0.05 vs. control.

**Figure 7.**
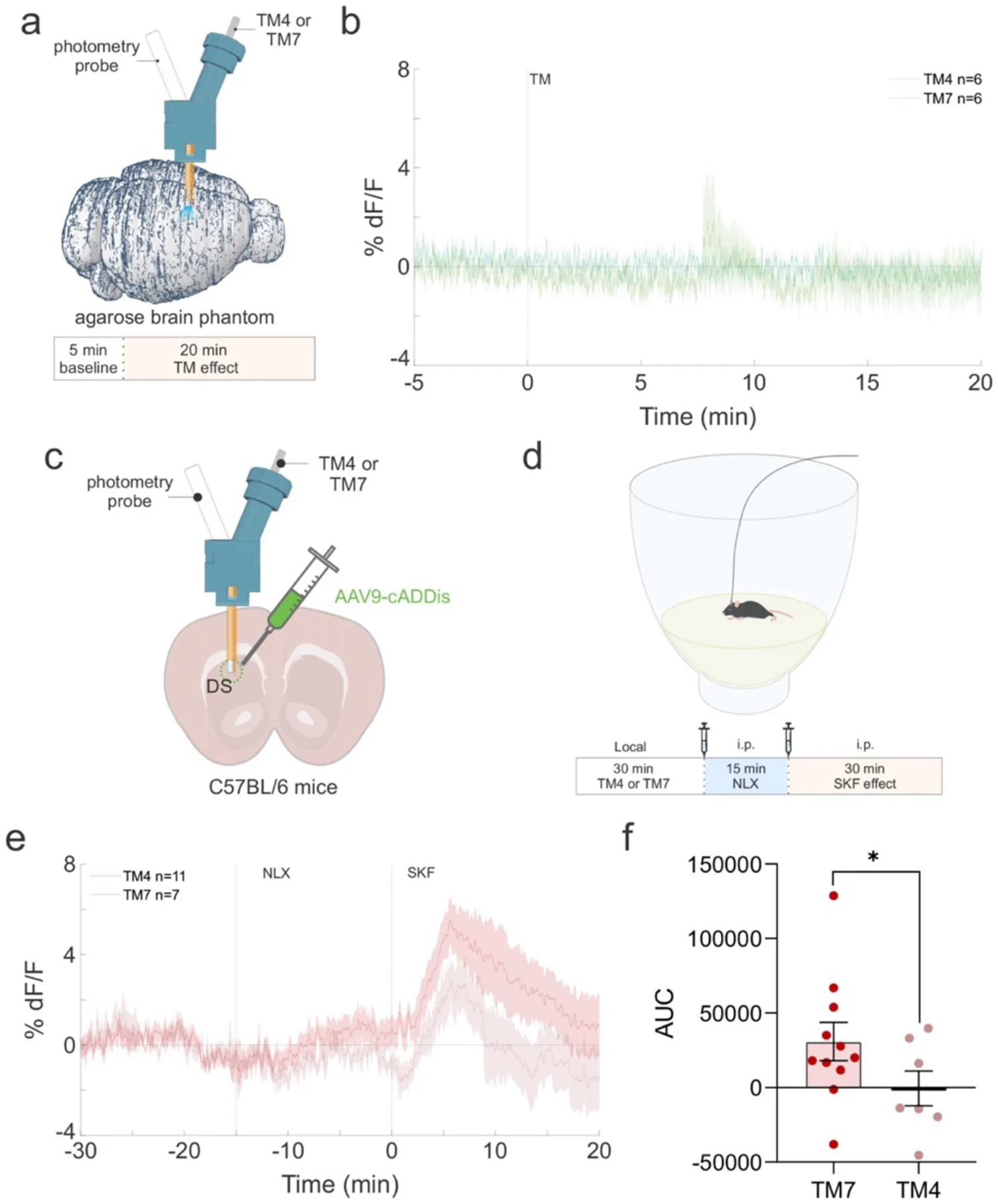
*In vivo* modulation of cAMP mediated by the MOR–D_1_R heteromer in the mouse dorsal striatum. (**a**) Experimental strategy using agarose phantom brains, a photo-fluidic probe, and fiber photometry to evaluate the autofluorescence of TM peptides. (**b**) Autofluorescence of TM4 and TM7 MOR peptides following local microinfusion into agarose phantom brains. (**c**) Schematic representation of viral cADDis microinfusion and photo-fluidic probe implantation in the dorsal striatum. (**d**) Experimental strategy to evaluate in vivo modulation of striatal cAMP mediated by the MOR–D_1_R heteromer using local microinfusion of MOR TM peptides. (**e**) Effect of local microinfusion of TM4 or TM7 MOR peptides on the naloxone (NLX)-mediated attenuation of SKF81297 (SKF)-induced increase in striatal cAMP. (**f**) Area under the curve (AUC) measured following systemic SKF administration after a 40-min local pretreatment with TM4 or TM7 MOR peptides and a 15-min systemic NLX pretreatment. Shaded areas (**b** and **e**) and error bars (**f**) represent SEM. Vertical dotted lines indicate the time of the systemic administration. Horizontal dotted lines indicate baseline fluorescence (set to 0%). Statistical significance was evaluated using unpaired *t*-test. P< 0.05 is denoted by *.

## Data Availability

All data that support the findings of this study are available from the corresponding author upon reasonable request.
